# Progress in Rectal Cancer Treatment

**DOI:** 10.5402/2012/648183

**Published:** 2012-08-30

**Authors:** Wim P. Ceelen

**Affiliations:** Department of Surgery, Ghent University Hospital, De Pintelaan 185, 9000 Ghent, Belgium

## Abstract

The dramatic improvement in local control of rectal cancer observed during the last decades is to be attributed to attention to surgical technique and to the introduction of neoadjuvant therapy regimens. Nevertheless, systemic relapse remains frequent and is currently insufficiently addressed. Intensification of neoadjuvant therapy by incorporating chemotherapy with or without targeted agents before the start of (chemo)radiation or during the waiting period to surgery may present an opportunity to improve overall survival. An increasing number of patients can nowadays undergo sphincter preserving surgery. In selected patients, local excision or even a “wait and see” approach may be feasible following active neoadjuvant therapy. Molecular and genetic biomarkers as well as innovative imaging techniques may in the future allow better selection of patients for this treatment option. Controversy persists concerning the selection of patients for adjuvant chemotherapy and/or targeted therapy after neoadjuvant regimens. The currently available evidence suggests that in complete pathological responders long-term outcome is excellent and adjuvant therapy may be omitted. The results of ongoing trials will help to establish the ideal tailored approach in resectable rectal cancer.

## 1. Introduction

Significant advancements have been made during the last decades in the treatment of rectal cancer. Once considered an incurable disease, treatment-related morbidity and mortality have decreased from 100% to less than 5% [1]. At the same time, the risk of locally recurrent disease, once seen in over 30% of patients and associated with a horrible fate, has been reduced to less than 5% in recent years. The progress in oncological outcome has led to the observation that, very recently, the long-term survival of rectal cancer is actually better than that of colon cancer [2]. This progress may be attributed to increased attention to surgical technique and to the introduction of combined modality therapy regimens. Nevertheless, several uncertainties persist regarding neoadjuvant therapy approaches, extent and technique of surgery, and selection for adjuvant therapy. Here, we highlight the currently accepted standard of care in the several steps of the patient's treatment trajectory. Also, we identify areas of uncertainty or controversy and identify important ongoing clinical studies aiming to resolve these issues.

## 2. Staging

The aim of staging procedures is twofold: first, to enable to tailor the therapeutic approach to the extent of locoregional as well as systemic disease identified and, second, to allow prognostic stage grouping and identification of those patients at high risk of recurrence. 

General staging includes measurement of carcinoembryonic antigen (CEA) and CT scan of chest and abdomen. Compared to colon cancer, the incidence of synchronous and metachronous pulmonary metastasis is notably higher in rectal cancer patients, a finding likely explained by the fact that venous drainage of the mid and lower rectum is systemic rather than portal [3–6]. 

Locoregional staging essentially comprises endoscopic ultrasound (EUS) and magnetic resonance imaging (MRI). Endoscopic ultrasound is readily available, inexpensive, and allows accurate staging of early cancers. The sensitivity and specificity of EUS in stage T1 and T2 tumours are >90% and >85%, respectively, while the accuracy of EUS to predict mesorectal nodal involvement is 70%–80% [7–9]. The disadvantages of EUS include its invasive nature and the inability to assess the circumferential resection margin (CRM).

Thin-section, T2-weighted, phased-array coil MRI of the pelvis allows excellent soft tissue spatial and contrast resolution. The MERCURY (Magnetic Resonance Imaging and Rectal Cancer European Equivalence) multicenter study group examined the ability of MRI to predict extramural invasion depth (EMD) by comparing imaging data with histopathological analysis of the resected specimens [10]. They found that, in an analysis of 295 patients, the mean difference between the MRI derived and histopathologically derived EMD was only −0.05 mm (95% confidence interval −0.49–0.40), resulting in actual equivalence between MRI and histological assessment of tumour spread. Similarly, the same group showed that MRI is highly accurate in predicting tumour involvement of the CRM and, thus, the likelihood of obtaining an R0 resection when primary surgery is used [11]. 

Mesorectal nodal metastasis represents one of the most powerful prognosticators in rectal cancer. Iconographic detection of nodal positivity is hampered by the considerable overlap in size between normal and cancer-invaded lymph nodes. When size criteria are combined with other morphological features such as mixed intranodal signal or border irregularity, the accuracy of MRI in predicting nodal status may reach 85% [12]. Accuracy may be further improved by using lymphotropic contrast agents such as ultrasmall superparamagnetic particles of iron oxide (USPIO), which are taken up by normal but not by tumour-invaded lymph nodes. Several authors have succeeded in considerably improving MRI accuracy of nodal staging with the USPIO ferumoxtran-10 [13–15]. Lambregts et al. reported the use of the macromolecular contrast agent gadofosveset (MS325), which is reversibly bound to plasma albumin [16]. They found that, using histolopathology as the standard reference, sensitivity and specificity of nodal staging improved from 76% and 82% to 80% and 97%, respectively, compared to standard MRI (*P* < 0.001).

## 3. Surgery

There is no doubt that the dramatic improvements in local control of rectal cancer during the past three decades are first and foremost due to improvements in surgical training and attention to technique [17–20]. The basic principles of total mesorectal excision (TME) are twofold: first, sharp dissection between the visceral and parietal layers of the mesorectal fascia and, second, complete excision of the mesorectum down to the pelvic floor in mid and low lying rectal cancer. The latter principle is based on the seminal observations by Heald and Quirke, who noted that the mesorectum may harbor tumour deposits up to 4 cm distal (caudal) to the lower edge of the luminal tumour [21–24]. In parallel, it was realized that the distal bowel resection margin may be safely reduced to less than 10 mm, and this finding, combined with novel strategies such as intersphincteric resection and coloanal anastomosis, has led to a significant increase in sphincter preserving procedures [25–29]. In patients with low lying tumours necessitating rectal amputation, the improvements in local control have been far less satisfying. This may be explained by an inherently different (namely, more invasive) tumour biology, but surgical factors were shown to play an important part. Indeed, when the mesorectal plane is dissected down to the pelvic floor, one inevitably gets very close to the tumour when it is located near the dentate line, a location where the mesorectum becomes very thin. Investigators from the Dutch Rectal Cancer Trial demonstrated that, compared to anterior resection, abdominoperineal resection resulted in a significantly higher risk of involved CRM (26.5% versus 12.6%, *P* < 0.001), a higher rate of tumour perforation, and worse survival [29]. The recent introduction of “cylindrical” resection, encompassing wide or complete removal of the levator muscle plate en bloc with the rectal amputation specimen, was shown to reduce the risk of CRM involvement, intraoperative perforation, and local recurrence [30–32]. In colon cancer, randomized trials have shown that, compared to open surgery, laparoscopic- or laparoscopy-assisted approaches are associated with significant functional advantages while cancer recurrence rates are similar [33, 34]. In rectal cancer, where preservation of the intact mesorectal envelope is of critical importance, the feasibility and safety of laparoscopic resection were suggested in several small trials [35]. The oncological equivalence of laparoscopic versus open rectal cancer surgery will need to be demonstrated by the ongoing prospective randomized trials: COLOR II, Japanese JCOG 0404, and ACOSOG Z6051 [36–38]. Preliminary results from the COLOR II trials were recently reported [39]. A total of 1103 patients were randomized to either laparoscopic or open rectal cancer surgery in a 2 : 1 ratio. No differences were observed in circumferential or distal margin, anastomotic leakage rate (8.8% versus 10% after laparoscopic versus open surgery, resp.; *P* = 0.63), or nodal count. However, the laparoscopic approach resulted in less blood loss, less analgesic use, quicker return of GI function, and shorter hospital stay. Conversion to open surgery was required in 16% of the patients allocated to laparoscopic surgery. Clearly, when confirmed by long-term oncological equivalence, the laparoscopic approach may be advised in selected patients. 

For most patients, avoidance of a permanent colostomy in low lying tumours is a concern. The introduction of long-term neoadjuvant chemoradiation followed by downsizing of the tumour allows to achieve sphincter preservation in many cases. In parallel, the technique of intersphincteric resection, colonic pull through, and manual coloanal anastomosis has gained popularity in low lying cancers. It has been shown that, in contrary to mesorectal distal tumour spread, infiltration of the rectal wall distally from the macroscopic lower tumour border is very uncommon and, consequently, a distal resection margin of 10 mm does not compromise R0 resection. Bujko and coworkers performed a meta-analysis of 17 studies reporting local recurrence rate according to distal resection margin (less than versus at least 1 cm) and found no statistically significant difference in local recurrence rate or overall survival [19]. It is clear, however, that functional results are potentially much worse after coloanal anastomosis and careful preoperative counseling is therefore mandatory, specifically in the elderly or those with preexisting impaired continence [29, 40]. 

## 4. Neoadjuvant Radiation and Combined**** Modality Therapy

Significant advances have been made in local control of advanced rectal cancer by combining surgery with either pre- or postoperative radiation therapy (RT). A meta-analysis based on individual patient data (IPD) published in 2001 showed that preoperative RT reduced the yearly risk of local recurrence with 46% (*P* = 0.00001), while postoperative RT reduced the risk with 37% (*P* = 0.002) [41]. Overall survival, however, was only marginally improved (62% versus 63%; *P* = 0.06). From a theoretical point of view, preoperative RT is associated with several advantages compared to the postoperative approach. First, RT will be more active in surgically undisturbed, well-oxygenized tissue. Second, only the preoperative approach may result in downstaging and downsizing effects. Third, postoperative RT administration may be hampered by compliance issues in the postoperative setting and may lead to increased radiation damage to the small bowel. Superiority of preoperative to postoperative chemoradiation was demonstrated by the German rectal cancer trial, showing that the preoperative approach resulted in improved local control and less toxicity, but no difference in overall survival was noted [42]. 

Importantly, it has been shown that protracting RT duration shifts the dose-response curve that describes control of subclinical pelvic tumour deposits to the right, resulting in a higher dose required to exert a similar reduction in pelvic relapse rate [43]. A recent meta-analysis and meta-regression related the biologically equivalent dose (BED) and fractionation schedule of preoperative RT to survival, local control, and sphincter preservation rate in rectal cancer [44]. It was found that, when a BED of more than 30 Gray is used, both short-term and long-term RT schedules were effective in improving local control and survival, while only a long-term schedule resulted in increased sphincter preservation. The Dutch randomized rectal cancer trial showed that preoperative short-term RT is effective even when standardized, quality-controlled TME surgery is routinely used [45]. The recently published 12-year update from this trial confirmed a significant reduction in local recurrence rate (5% after RT followed by surgery versus 11% in the surgery alone group, *P* < 0.0001) [46]. Of note, the results of this trial also showed that, in patients with involved resection margins, preoperative RT cannot prevent local recurrence [47]. 

There are sound theoretical arguments for combining RT with chemotherapy. First, several chemotherapeutic agents act as radiosensitizers and will enhance the pathological effects of RT. Second, early incorporation of chemotherapy might address concurrent systemic disease. Several prospective randomized trials have compared preoperative RT alone with preoperative chemoradiation (CRT) in locally advanced rectal cancer (Table 1). The results of the completed trials and ongoing studies with preliminary results may be summarized as follows: compared to RT alone, preoperative CRT improves pathological response and local control but is associated with more pronounced treatment-related toxicity. In addition, CRT does not benefit sphincter preservation rate or long-term survival rate [48]. Debate persists regarding the place of short course RT (SCRT) schedules (usually 5 × 5 Gray) followed by immediate surgery. Arguments in favour include (1) the fact that SCRT has been tested and found to be effective in multiple randomized trials, (2) convenience for the patient, and (3) the similarity in long-term outcome when compared with long-term (C)RT schedules. Disadvantages of SCRT include significant early and delayed toxicity (including secondary malignancies) observed in the Swedish and Dutch rectal cancer trials, and the inability to achieve downstaging and downsizing when immediate surgery (within 5–10 days) is performed [49–52]. It should be noted, however, that with modern conformal RT delivery techniques the differences in toxicity between SCRT and long schedule RT schedules seem minimal. An important question is whether a longer waiting period after SCRT would achieve pathological downstaging to the extent observed with long-term schedules. The interim results from the trial reported by Latkauskas et al., who randomized patients to receive either SCRT or CRT and included a six weeks waiting period in both groups, demonstrated a far superior pathological response in the group who underwent chemoradiation [53]. Additional answers are awaited in this regard from the ongoing Stockholm III trial, which randomizes patients to receive either SCRT with immediate surgery, SCRT with delayed (after 4–8 weeks) surgery, or long course radiotherapy (25 × 2 Gray) with delayed surgery [54]. 

Incorporation of additional chemotherapy agents in preoperative regimens, aiming at further enhancing pathological response whilst possibly improving overall survival, seemed a rational step. Several phase III trials have been initiated incorporating oxaliplatin, an agent which is active in the adjuvant and palliative setting, in CRT regimens. From four of these trials, interim data are available (Table 2). The first results of the PETACC-6 trial, which randomizes patients to preoperative RT (50.4 Gray in 25 fractions) with capecitabine alone or with capecitabine + oxaliplatin (50 mg/m^2^), are awaited. The available results from the other four trials suggest that the expectation of an increased pathological response by adding oxaliplatin to the CRT regimen was not fulfilled; only in the German CAO/ARO/AIO-04 trial a significantly different pathological complete response rate was observed in favour of the arm containing oxaliplatin [55]. Moreover, incorporation of oxaliplatin resulted in significantly higher rates of grade 3 and 4 treatment-related toxicity in three out of four trials that have reported on these data [56–58]. 

There is a sound theoretical rationale to combine preoperative CRT with each of the targeted agents approved for use in metastatic colorectal cancer (mCRC): cetuximab, panitumumab, and bevacizumab. Epidermal growth factor receptor (EGFR) signaling is associated with proliferation, invasiveness, and metastasis. Several arguments suggest a potential synergism between EGFR inhibition and RT. First, EGFR tyrosine kinase activity is increased in cancer cells in response to RT, and addition of exogenous EGF can induce radioresistance in vitro [70]. Second, elevated levels of EGFR expression are an independent adverse prognostic factor in rectal cancer patients [71]. Numerous phase I/II trials have studied incorporation of cetuximab in preoperative CRT schedules. The early results regarding pathological response (pCR rate) are disappointing [72–75]. Interestingly, KRAS mutation, known to be an adverse predictive and prognostic marker in mCRC patients treated with EGFR inhibitors, is less frequent in rectal cancer and does not convey the same predictive information [76, 77]. The recently reported randomized EXPERT-C trial allocated high-risk rectal cancer patients to four cycles of capecitabine/oxaliplatin (CAPOX) followed by capecitabine CRT, TME surgery, and adjuvant CAPOX or the same regimen plus cetuximab [78]. The primary endpoint was complete (pathological or radiological) response in KRAS/BRAF wild-type tumours. Addition of cetuximab did not affect complete response rate or progression free survival, although it did improve radiological response and overall survival (hazard ratio 0.07–0.99; *P* = 0.034). 

In parallel, efforts have started to combine preoperative CRT regimens with the antiangiogenic agent bevacizumab. Therapy directed against the vascular endothelial growth factor (VEGF) causes “normalization” of the tumour vascular bed, that is, the return to a functionally and morphologically less deficient microvascular network [79, 80]. This phenomenon is accompanied by increased oxygenation and a decrease in tissue interstitial fluid pressure (IFP). Moreover, it was shown in vitro that RT induces tumour VEGF expression and protects tumour blood vessels from RT-mediated cytotoxicity [81]. Numerous phase I/II trials have studied the integration of bevacizumab into CRT regimens [82–91]. The addition of bevacizumab appears to enhance pathological response rates but is associated with increased treatment related and postoperative complications including wound dehiscence, bowel perforation, and bleeding [92, 93]. Of note, several imaging (blood flow, perfusion) and molecular biomarkers (soluble VEGF receptor, VEGF, placental derived growth factor, IL-6, and circulating endothelial cells) were shown to correlate with outcome after bevacizumab-based combined modality therapy [91].

## 5. Novel Chemotherapy Treatment Strategies

A consistent finding of the myriad of neoadjuvant radiotherapy containing trials is that hardly any progress has been made in improving overall survival. Consequently, strategies are investigated that aim at delivering more efficient systemic therapy early in the course of therapy. These strategies include induction chemotherapy followed by CRT, and CRT followed by consolidation chemotherapy in the waiting period to surgery. Intensive preoperative chemotherapy not only has the potential to eradicate subclinical metastatic disease but also avoids the inherent compliance problems of postoperative chemotherapy. Several phase II trials have now generated results of neoadjuvant chemotherapy (NACT) followed by CRT (Table 3). Although the survival data seem promising, the two randomized phase II studies comparing upfront CRT with NACT followed by CRT failed to demonstrate improvements in either pCR rate or R0 resection probability [94, 95]. Investigators from the AVACROSS study, who incorporated bevacizumab in both the NACT and CRT regimens, found an impressive pCR rate 36% while 98% of patients were able to undergo an R0 resection [96]. However, with a reoperation rate of 24% and anastomotic leakage rate of 17%, surgical morbidity appears a significant problem when intensifying preoperative regimens with antiangiogenic agents.

An alternative approach is to administer “consolidation” chemotherapy during the waiting period to surgery. Preliminary data were provided by Habr-Gama et al., who treated rectal cancer patients with 54 Gy of RT with 5-FU/leucovorin followed by an additional three cycles of 5-FU/leucovorin; clinical response was assessed ten weeks after completion of CRT [97]. Fourteen out of 29 patients (48%) were found to have a complete clinical response, while an additional 17% had a ypT0 stage after local excision. Garcia-Aguilar and coworkers in a nonrandomized phase II trial compared 5-FU based neoadjuvant CRT with a regimen that added two cycles of FolFox in patients who had a clinical response four weeks after CRT [98]. They found a modest increase in pCR rate (25% versus 18%); whether this is an effect of prolonging the waiting period from 6 to 11 weeks or of the consolidation chemotherapy cannot be discerned. Important answers will be provided by the ongoing phase III RAPIDO trial (NCT01558921), which will randomize high-risk rectal cancer patients to undergo either neoadjuvant SCRT followed by six cycles of CapOx, or standard neoadjuvant chemoradiation. Intriguingly, recent reports suggest that modern neoadjuvant combination chemotherapy may result in effective downstaging even without any radiotherapy. Schrag reported on a group of patients with resectable rectal cancer who received induction chemotherapy (FolFox with bevacizumab) and were planned to undergo additional CRT or immediate surgery depending on clinical regression [99]. Interestingly, all of the 29 treated patients underwent surgery without CRT and an impressive pCR rate of 27% was noted. No local recurrences were observed, and three patients (10%) developed distant metastases (all pulmonary).

## 6. Adjuvant Chemotherapy 

At present, it is unclear how patients who underwent neoadjuvant combined modality therapy should be selected for adjuvant chemotherapy. In contrast to colon cancer, there are no randomized adjuvant therapy trials in rectal cancer. A recent Cochrane meta-analysis, based on data concerning patients from trials including both colon and rectal cancer, showed that 5-FU-based adjuvant chemotherapy following curative resection of rectal cancer confers a significant advantage in terms of overall (HR = 0.83, 95% CI: 0.76–0.91) and disease-free (HR = 0.75, 95% CI: 0.68–0.83) survival although significant heterogeneity between trials was noted [118]. Unfortunately, due to limitations of the source data, the authors were unable to identify those TNM stages that benefit most from adjuvant chemotherapy. One of the included trials in the Cochrane meta-analysis assigned colon and rectal cancer patients at low risk of recurrence (90% stage II) to either adjuvant 5-FU/folinic acid or observation [119]. Interestingly, planned subgroup analysis showed that the relative risk of recurrence within 2 years was lower in rectal cancer (29% of patients) than in colon cancer patients (RR 0.38–0.89 and 0.54–0.92, resp.). Based on these data, it seems reasonable to offer adjuvant chemotherapy to rectal cancer patients based on similar criteria as in colon cancer. However, matters are complicated by the fact that nowadays most rectal cancer patients are treated with neoadjuvant regimens, and uncertainty persists regarding the benefit of adjuvant therapy in patients who have a complete or near complete pathological response. On the one hand, patients with a pathological complete response have a significantly better outlook. In a recent pooled analysis using individual patient data, 5-year disease-free survival was 83.3% for patients with pCR and 65.6% for those without pCR (HR 0.44, 95% CI 0.34–0.57; *P* < 0.0001) [120]. On the other hand, (unplanned) subgroup analysis of the four-arm EORTC 22921 trial suggested that, while adjuvant chemotherapy did not affect outcome in the whole study population irrespective of whether preoperative RT or CRT was administered, adjuvant chemotherapy significantly improved survival in ypT0-2 patients (but not in ypT3-4) [121]. However, the generalizability of this finding has been questioned on methodological grounds; moreover, in none of the other three randomized trials exploring adjuvant chemotherapy in patients who received preoperative (C)RT has any benefit been detected [119, 122–124]. Clearly, therefore, the role of 5-FU-based adjuvant therapy in patients who received chemotherapy containing neoadjuvant regimens remains undefined. Only one prospective randomized trial (SCRIPT, Simply Capecitabine in Rectal cancer after Irradiation Plus TME surgery) by the Dutch colorectal cancer group is testing adjuvant chemotherapy versus observation in rectal cancer patients who received neoadjuvant RT or CRT. 

## 7. Innovation in Radiation Techniques

One of the insights gained in the radiobiology of rectal cancer over the past years is that a biologically effective dose (BED) of at least 30 Gray needs to be administered in order to affect the risk of local recurrence [43, 125]. The pathological complete response rates obtained with modern chemoradiation schedules are in the order of 10–15%. Efforts have been directed to enhance the therapeutic index of radiotherapy by increasing conformity to the target tissue. Highly conformal techniques such as intensity modulated radiotherapy (IMRT), intensity modulated arc therapy (IMAT), and tomotherapy were shown to result in clinically significant reductions in GI toxicity by limiting the dose delivered to the small bowel [126–130]. An interesting option is endocavitary contact radiotherapy, which may completely sterilize early (T1N0 and T2N0) rectal tumours and was shown to result in significant improvements in pCR and sphincter preservation rates when combined with external beam radiotherapy [131–134]. The technique had fallen into disuse over the last years due to the fact that the apparatus was no longer produced. Recently, however, another manufacturer has brought a novel machine on the market (Papillon 50, Ariane Medical Systems, Derby, UK). Several international trials (Contact Endoscopic Microsurgery, CONTEM 1–3) were recently initiated using a combination of contact endocavitary RT with transanal microsurgery, CRT, or standard TME in patients with T1, T2, or early T3 rectal tumours [135, 136]. 

## 8. Organ Preservation in Rectal Cancer

In analogy to current practice in cancer of the anal canal, definitive chemoradiation combined with local excision or without further surgery is under active scrutiny [137, 138]. There are, nevertheless, several major hurdles to be taken before the concept of organ preservation will gain wide acceptance. First, clearly the adenocarcinoma of the rectum is a much less radiosensitive tumour, and pathological response rates tend to be even lower in general practice compared to what is achieved in the setting of clinical trials [139]. Second, clinical as well as endoscopic and iconographic restagings after CRT are notoriously unreliable, and even post-CRT biopsies are inaccurate in predicting sterilization of both the tumour and the mesorectal nodes [140–143]. Clearly, therefore, a mere “wait and see” approach should currently only be considered in patients unfit for or refusing surgery. There may be a role for local excision in patients who have a substantial clinical response after chemoradiation. Several retrospective reports and small prospective trials have shown impressive pCR rates in early rectal cancers, while both local control and long-term survivals seem excellent (Table 4). Although local excision techniques certainly present less surgical risks compared to resectional procedures, it should be emphasized that they may carry their own specific morbidity such as significant pain after TEM [144]. Also, the results of these preliminary data based on a highly selected population need to be confirmed in prospective controlled trials. The multicenter CARTS trial in The Netherlands will investigate the feasibility of neoadjuvant CRT (25 fractions of 2 Gy with concurrent capecitabine) followed by transanal endoscopic microsurgery (TEM) in patients with clinical stage T1-3, N0 rectal cancer below 10 cm from the anal verge [145]. The French multicenter Groupe de Recherche Chirurgicale sur le Cancer du Rectum (GRECCAR) 2 trial (NCT00427375) will treat rectal cancer patients with a tumor 4 cm or less in diameter with neoadjuvant CRT. After a 6–8-week waiting period, patients in whom the tumour has downstaged to 2 cm or less will be allocated the either local excision or TME. A polish multicenter trial (NCT00738790) will randomize patients with cT1-3, N0 rectal cancer patients to either short course RT (5 × 5 Gy with a 4 Gy boost after 1 week) or CRT followed by local excision after 6 weeks. A Spanish trial (NCT01308190) will compare in a randomized trial primary TME with CRT followed by local excision in patients with clinically staged T2 or superficial T3 low rectal cancer.

Clearly, the adaptation of local resection strategies will depend on our ability to predict the extent of pathological response using clinical, molecular, and imaging biomarkers. Novel imaging techniques such as diffusion-weighted magnetic resonance imaging hold promise in the identification of responders [146–151]. Similarly, gene expression profiling has recently been successfully used to predict pathological response to CRT. Ghadimi et al., using cDNA material obtained during the German CAO/ARO/AIO-94 trial, found that a 54 gene signature correctly predicted response in 83% of patients [152]. Similar results were reported by Brettingham-Moore and coworkers, although this group was unable to validate previously published gene expression based classifiers in their patient cohort, illustrating the difficulty in comparing and generalizing the use of these classifiers due to the high dimensionality of the data [153]. Several other molecular, genetic, and chromosomal biomarkers of response to CRT in rectal cancer have been identified, and these biomarkers are increasingly integrated into clinical trial design [154–160]. 

## Figures and Tables

**Table 1:** Prospective randomized comparisons between radiotherapy alone and chemoradiation.

Study	Tx	*N*	RT dose (Gy) Total per fraction	Chemo	pCR	SPS	LR at 5 years	OS at 5 years	DFS at 5 years	Incidence of distant metastases
EORTC (Boulis-Wassif et al.) [59]	CRT	126	34.5/2.3	5-FU	4.8%	10.5%	15.1%	54%	—	30% overall
	RT	121	34.5/2.3	—	2.5%	5%	14.9%	41.3%	—	
EORTC 22921 (Bosset et al.) [60–62]	CRT	506	45/1.8	FUFA	13.7%	52.8%	8.7%*	65.8%	56.1%	34.4% overall
	RT	505	45/1.8	—	5.3%	50.5	17.1%*	64.8%	54.4%	
FFCD 9203 (Gérard et al.) [63, 64]	CRT	375	45/1.8	FUFA	11.4%	52.7%	8.1%	67.4%	59.4%	—
	RT	367	45/1.8	—	3.6%	51.8%	16.5%	67.9%	55.5%	—
Polish trial (Bujko et al.) [65–67]	CRT	157	50.4/1.8	FUFA	16%	55.4%	14.2%	66.2%**	55.6%**	34.6%
	RT	155	25/5	—	1%	56.1%	9%	67.2%**	58.4%**	31.4%
GRECCAR I [68]	CRT	101	45/1.8	FUFA	12.5%	86%	5%	—	—	—
	RT	106	45 + 18	—	7%	83%	6%	—	—	—
Australian Intergroup [69]	CRT	163	50.4/1.8	FUFA	—	—	4.4%^#^	70%	69%	—
	RT	163	25/5	—	—	—	7.5%^#^	74%	72%	—
Lithuanian study [53]	CRT	46	50/1.8−2	FUFA	13.1%	69.6%	—	—	—	—
	RT	37	25/5 (delayed surgery)	—	2.7%	70.3%	—	—	—	—

EORTC: European Organisation for the Research and Treatment of Cancer; FFCD: Fédération Francophone de la Cancérologie Digestive; RT: radiotherapy; pCR: pathological complete response; SPS: sphincter preserving surgery; LR: local recurrence; OS: overall survival; DFS: disease free survival; *in the groups not receiving postop chemotherapy (4 arm trial); **4-year data; ^#^3-year data actuarial survival.

**Table 2:** Interim results of randomized trials incorporating oxaliplatin in preoperative chemoradiation regimens.

Study	Treatment	*N*	RT dose (Gy) Total/per fraction	pCR	SPS
STAR01 [56]	5-FU CIV	379	50.4/1.8	16%	80%
	5-FU CIV + OX 60 mg/m^2^	368	50.4/1.8	16%	82%
ACCORD 12/0405-Prodige 2 [57]	CAP 800 mg/m^2^	299	45/1.8	13.9%	75%
	CAP 800 mg/m^2^ + OX 50 mg/m^2^	299	50/1.8	19.2%	75%
CAO/ARO/AIO-04 [55]	5-FU; adj 5-FU	637	50.4/1.8	13.1%	88.1%
	5-FU + OX 50 mg/m^2^; adj FolFox6	628	50.4/1.8	17.6%	87.8%
NSABP R-04 [58]	5-FU CIV ± OX 50 mg/m^2^	719	50.4/1.8	18.8%	61.2%
	CAP 825 mg/m^2^ ± OX 50 mg/m^2^	707	50.4/1.8	22.2%	62.7%

CAP: capecitabine; RT: radiotherapy; pCR: pathological complete response; SPS: sphincter preserving surgery.

**Table 3:** Neoadjuvant chemotherapy followed by chemoradiation in locally advanced rectal cancer.

	Study Regimen			*N*	int(*w*)	SPS	pCR	DFS	OS
	Neoadjuvant	CRT	Adjuvant						
Chau et al. [100]	5-FU, MMC (7)	50.4 Gy with 5-FU	5-FU, MMC (7)	36	6	56%	3%	1 y: 72.1%	2 y: 70.3%
EXPERT [101]	CAP (1000), OX (130)	54 Gy with CAP (825)	CAP (1250)	105	6	63%	20%	3 y: 68%	3 y: 83%
GCR3 [94]	—	50.4 Gy with CAP (825)	CAP (2000), OX (130)	52	5-6	—	13%	1.5 y: 82%	1.5 y: 89%
	CAP (2000), OX (130)	50.4 Gy with CAP (825)	—	56	5-6	—	14%	1.5 y: 76%	1.5 y: 91%
BGDO [95]	—	45 Gy with 5-FU CIV	—	29	6–8	67%	27%	—	—
	5-FU, OX (100)	45 Gy with 5-FU CIV	—	28	6–8	100%	25%	—	—
Schou et al. [102]	CAP(2000), OX (130)	54 Gy + CAP (1650)	None	84	6	—	25%	5 y: 63%	5 y: 67%
AVACROSS [96]	CAP (1000), OX (130), bev	45 Gy + CAP (825) + bev	CAP + OX	47	6–8	60%	36%	—	—
Dipetrillo et al. [83]	5-FU, OX (85), bev	50.4 Gy + OX (50) + bev	5-FU + OX + bev	26	4–8	76%	20%	4 y: 65%	4 y: 96%
EXPERT-C [78]	CAP (1700), OX (130)	50.4 Gy + CAP (1650)	CAP + OX	81	4–6	73%	15%	HR: 0.3–2.16	HR: 0.07–0.99
	CAP (1700), OX (130), cet	50.4 Gy + CAP (1650) + cet	CAP + OX + cet	84	4–6	73%	14%		

CAP: capecitabine; MMC: mitomycin C; OX: oxaliplatin; cet: cetuximab; bev: bevacizumab; RT: radiotherapy; pCR: pathological complete response; SPS: sphincter preserving surgery; OS: overall survival; DFS: disease free survival; int: interval in weeks.

**Table 4:** Results of local excision following neoadjuvant (C)RT.

Study	Year	*N*	cT stage	%cN+	(C)RT	LE technique	pCR	LR	OS	DFS
Garcia-Aguilar et al. [103]	2012	77	T2	0%	50.4–54 Gy with CAPOX	TAE, TEM	44%	—	—	—
Yeo et al. [104]	2010	11	T3	45%	50.4 Gy with 5FU, CAP, or CapIri	TAE	72%	9%	88.9% at 5 y	81.8% at 5 y
Callender et al. [105]	2010	47	T3	27.6%	45–52.5 Gy with 5FU	87% TAE, 13% Kraske	49%	10.6 at 10 y	74% at 10 y	76% at 10 y
Kundel et al. [106]	2010	20			50.4–54 Gy with 5FU	TAE	70%	0%	100% at 5 y	100% at 5 y
Bujko et al. [107]	2009	31	T1–T3	0%	5 × 5 Gy	39% TEM, 57% TAE,	35%	7%	—	—
		13			55.8 Gy with 5FU	4% Kraske	54%			
Huh et al. [108]	2008	9	T2,T3	33%	45–50.4 Gy with oral 5FU	TAE	44%	11% at 10 y	89% at 10 y	78% at 10 y
Nair et al. [109]	2008	44	T2,T3	25%	50.4 Gy with 5FU	TAE (11% salvage)	57%	16%	81% at 5 y	—
Guerrieri et al. [110]	2008	196	T2,T3	0%	50.4 Gy, some with 5FU	TEM	17%	4%	—	95% (T2), 87% (T3) at 3 y
Park et al. [111]	2007	7	T2,T3	0%	45 Gy with 5FU	TAE	43%	0%	No events	No events
Caricato et al. [112]	2006	8	T2–T4	25%	45 Gy with 5FU + cisplatin	62% TEM, 38% TAE	37%	12%	No events	No events
Bonnen et al. [113]	2004	26	T3	4%	45–52.5 Gy with 5FU	88% TAE, 12% Kraske	54%	6% at 5 y	86% at 5 y	80% at 5 y
Ruo et al. [114]	2002	10	T2,T3	—	36–50.4 Gy, with 5FU	TAE	30%	20%	78% at 2 y	—
Schell et al. [115]	2002	11	ycT0,T1	27%	45 Gy with 5FU	TAE	73%	0%	No events	1 pulm. Metastasis
Kim et al. [116]	2001	26	T2,T3	27%	45 Gy with 5FU	TAE	65%	4%*	—	—
Mohiuddin et al. [117]	1994	48	T1–T3	—	55 Gy	TAE	37%	11%	83% at 5 y	—

RT: radiotherapy; LE: local excision; TAE: transanal excision; TEM: transanal endoscopic microsurgery; pCR: pathological complete response; SPS: sphincter preserving surgery; OS: overall survival; DFS: disease-free survival; LR: local recurrence.

*In a patient with pPR refusing additional surgery.
